# Nutritional Composition, Fatty Acids Profile, Mineral Content, Antioxidant Activity and Acute Toxicity of the Flesh of *Helix aspesra* Müller

**DOI:** 10.3390/molecules28176323

**Published:** 2023-08-29

**Authors:** Marouane Aouji, Hamada Imtara, Amine Rkhaila, Bouchra Bouhaddioui, Ahmad Alahdab, Mohammad Khalid Parvez, Mohamed Saleh Alzahrani, Lalla Aicha Lrhorfi, Rachid Bengueddour

**Affiliations:** 1 Laboratory of Natural Resources and Sustainable Development, Department of Biology, Faculty of Sciences, Ibn Tofail University, BP 133, Kenitra 14000, Moroccoalrhorfi_com@yahoo.fr (L.A.L.); rachidbengueddour@yahoo.fr (R.B.); 2 Faculty of Arts and Sciences, Arab American University Palestine, Jenin P.O. Box 240, Palestine; hamada.tarayrah@gmail.com; 3 Laboratory of Plant, Animal and Agro-Industry Productions, Department of Biology, Faculty of Sciences, Ibn Tofail University, BP 133, Kenitra 14000, Morocco; 4 Institute of Pharmacy, Clinical Pharmacy, University of Greifswald, Friedrich-Ludwig-Jahn-Street 17, 17489 Greifswald, Germany; 5 Department of Pharmacognosy, College of Pharmacy, King Saud University, Riyadh 11451, Saudi Arabia; mohkhalid@ksu.edu.sa (M.K.P.); mohalzahrani@ksu.edu.sa (M.S.A.)

**Keywords:** *H. aspersa* Müller, flesh, fatty acid, essentials elements, nutrients elements, antioxidant activity, acute toxicity, lethal dose 50

## Abstract

Humans consume snail flesh as part of their diet. To assess its nutritional value and toxicity, chemical analyses were conducted to confirm the presence of protein, total and reduced carbohydrates, fat, fatty acid composition and mineral components. Furthermore, an acute toxicity study was carried out to determine the safety of *Helix aspersa* Müller snail flesh. *H. aspersa* Müller snail flesh exhibits a high nutritional content, a good ω3/ω6 ratio and higher levels of unsaturated fatty acids. Various minerals have been found in the flesh of *H. aspersa* Müller. Around 76.91 kcal, or 3.84% of the energy of a daily meal of 2000 kcal, are present in 100 g of this flesh. The evaluation of the antioxidant capacity indicated that the flesh’s extracts contained a large quantity of antioxidant biomolecules. Administration of the aqueous extract of *H. aspersa* Müller flesh didn’t cause death in laboratory rats, indicating that the lethal dose 50 is greater than 2000 mg·kg^−1^ body weight. The consumption of the flesh of *H. aspersa* Müller is highly recommended for human consumption due to its high concentration of nutrients and essential elements, as well as unsaturated fats, and due to its safety.

## 1. Introduction

Inexpensive natural resources are used for their medicinal virtues in the prevention or treatment of a variety of pathologies. Most people believe that natural products are non-harmful and safe [1]. Natural extracts have become increasingly popular in recent years as functional or therapeutic sources [2]. However, as the use of these natural extracts increases, questions have been raised about their safety. It is essential to assess the safety of natural extracts in order to make the best use of them. To verify their efficacy and quality, thorough testing must be carried out [3]. The safety of chemicals is determined using acute and sub-acute toxicity tests, and the results are used to identify hazards and control risks associated with the creation, processing, and use of these products [4]. Assessing the safety of natural extracts will also provide scientific data on their various applications.

*Mollusks* are the second-largest branch of the animal kingdom, with around 100,000 species [5]. The gastropod class includes more than 35,000 species of land snails, which are found worldwide [6]. In many countries, they have been eaten for centuries. Among them, the *Helicidae* genera are the most widely consumed [7].

Snail products are a highly nutritious meal, and snails have long been an important source of food for humans [8]. They are eaten by people worldwide, rich and poor, urban and rural. Snail consumption has been associated with a number of health benefits [9,10,11,12,13]. The main nutritional benefits of snail flesh reside in its high protein content, its low-calorie content due to its low lipid and carbohydrate content, its high concentration of vital minerals, and the presence of B-complex and fat-soluble vitamins [14]. The presence of necessary amino acids and unsaturated fats is a further advantage [15]. In terms of nutritional value, the main advantage of snail flesh is that its essential ingredients are all likely to improve consumer health [14]. Its consumption can be considered an alternative to consuming other higher-calorie foods, from a dietetic perspective [14,15]. Ajayi et al. [16] have shown that snail flesh is particularly rich in iron and protein. The low fat and cholesterol content of snail flesh makes it a powerful means of preventing vascular disease. Snail flesh is also rich in polyunsaturated fatty acids [17]. As a result, snail flesh can be considered a high-protein, low-fat food [18], which explains its widespread consumption in many European countries [13].

Murphy [19] has stated that *H. aspersa* flesh provides several nutrients necessary for a healthy, balanced diet. In addition, snail flesh is beneficial to health, as it contains little cholesterol and is rich in minerals [20]. The mineral composition of snails is closely linked to location, diet, seasons and life cycles, in addition to the variations inherent in different species. The biochemical composition of the tissues of a species or individual is influenced by variable genetic and physiological factors which in some cases affect nutritional content [10,12].

Live, processed, frozen, dried or canned snails can all be purchased. Because “slimy texture” is usually associated with “disgusting”, many people are reluctant to eat whole snails [21]. Snail flesh powder, which can be used in a variety of dishes and enhances the snail’s flavor and, consequently, its acceptance by the consumer, is obtained by dehydrating and grinding the snail’s flesh. The dried and powdered version of snail flesh can also increase its shelf life and facilitate product distribution and management [22].

Direct and mineral analysis of snails has been reported [14]. The garden snail *H. aspersa* Müller is one of the most widely consumed land snail species [23]. It is the species preferred by the heliciculture industry, as it is best suited to commercial production due to its reproductive capacity, rapid life cycle, flavor, flexibility and ability to adapt to different conditions and geographical locations [24].

This high level of consumption raises the question of the nutritional potential and toxicity associated with the consumption of this food. For this reason, the main objective of this study was to examine the nutritional and mineral content of *H. aspersa* Müller flesh. Several indicators, including proteins, lipids, carbohydrates, ash, fatty acid profile and mineral elements, are determined, along with the chemical composition of the aqueous extract of *H. aspersa* Müller flesh and the study of its antioxidant activity, as well as an investigation of the acute toxicity associated with the consumption of this species in rats.

## 2. Results and Discussion

### 2.1. Proximate Analysis

#### 2.1.1. Moisture Content, Dry-Matter Content and Acidity Measurement

The analyses of the results reveal a water content of 84.24 ± 1.21% in the flesh of *H. aspersa* Müller (Table 1). This finding partially concurs with the conclusions of Çağıltay et al. [25], who observed an average water content of 82.5% for *H. aspersa*. However, further investigations carried out on *H. aspersa aspersa* and *H. aspersa* maxima by Milinsk et al. [26] and Gomot [20], respectively, revealed proportions of 83.7% and 87.4% in terms of water content.

It is important to mention that other species have also been evaluated for their water content. In the case of *H. pomatia*, a rate of 80.8% [12] has been found. Furthermore, quantities of 84.2% and 84.0% have been detected in *H. pomatia* and *H. lucorum*, respectively [20]. Variations are more apparent in the results obtained for *Cernuella virgata*, with slightly lower water contents ranging from 77.3% to 79.1% [10]. In contrast, water contents for African species ranged from 73.7% to 79.1% [11]. The intrinsic variability that characterizes these organisms, and their adaptation to particular environments, is underlined by this diversity of water contents within different species.

The amount of moisture in a snail species is strongly linked to the environment in which it lives and its natural habitat, including temperature and humidity, which explains the differences observed between species [20,27]. The composition of snail meat reveals a preponderance of water, generally ranging from 70 to 90%, with an average value often close to 80%.

Dry-matter content after complete drying was measured at 17.62 ± 0.74% for *H. aspersa* Müller flesh. These results are consistent with those of Caetano et al. [27], who also found similar dry-matter levels for *O. lactea* and *T. pisana*, at 16.5% and 22.6%, respectively.

Continuing the analysis, the titratable acidity of the snail meat was determined to be 0.45 ± 0.01%. In addition, a slightly alkaline pH was established, at 8.65 ± 0.07. According to Newar and Ghatak [28], this alkalinity may be due to the predominance of alkaline groups within protein and carbohydrate components, such as amino groups.

#### 2.1.2. Protein, Total Sugar and Fat Content

The average protein content of *H. aspersa* Müller flesh was 10.33 ± 0.21 g·100 g^−1^ DM. The results concur with those of Gomot’s study [20], in which samples of different species showed proportions such as *H. aspersa* maxima (7.2 g·100 g^−1^), *H. aspersa aspersa* (10.5 g·100 g^−1^), *H. lucorum* (10.8 g·100 g^−1^) and *H. pomatia* (10.7 g·100 g^−1^). Previous research has also confirmed the consistency of these data, with a protein content of 12.9% for *H. aspersa*, as well as a protein content of 9.6% for *O. lactea* and 11.4% for *T. pisana*, as reported by Caetano et al. [27]. However, higher levels have been highlighted, such as the protein contents of *Cernuella virgate*, according to Sando et al. [10], as well as values for *A. marginata* (19.5%) and *A. achatina* (17.3%) [11].

Carbohydrate results showed a total sugar of 2.88 ± 0.04 g·100 g^−1^ SM. According to several nutritional studies, the total amount of carbohydrates is less than 5%. Existing research on various species has provided diverse results, with *H. pomatia* at a total sugar of 1.3 g·100 g^−1^ [12], while *A. marginata* and *A. achatina* have 1.8 and 3.0 g·100 g^−1^, respectively [11]. There are higher values, such as 7.3% in *A. fulica* [11] and ranges from 7.1% to 10.0 g·100 g^−1^ in *H. aspersa* maxima [26] and 1.0% to 2.9% in *Cernuella virgata* [10]. In comparison, *O. lactea* and *T. pisana* range from 2.0% to 4.3% [27].

Our results show a fat content of 2.55 ± 0.00%. The results of Milinsk et al. [26] on *H. aspersa* maxima are consistent with these figures. Other research has shown a variety of variations, ranging from 0.41% for *H. pomatia*, according to Özogul et al. [12], to values close to 0.58% for *H. aspersa*, according to Çağıltay et al. [25]. However, the results of Gomot et al. [20] range from 1.1% to 1.2%, suggesting differences within the same species. Caetano et al. [27] also observe a range of 1.3% to 3.2%, while *A. marginata*, *A. achatina* and *A. fulica* have fat proportions of 1.6% to 2.6%, according to research by Babalola and Akinsoyinu [11]. These variations can be caused by the snail’s diet and habitat, offering elements to explain these notable differences.

### 2.2. Mineral Content

The total ash concentration measured after incineration was 1.89 ± 0.02%, showing that *H. aspersa* Müller contains a significant quantity of minerals. Similar findings have been reported in the literature (*H. aspersa*, 1.1% [25], mineral content in *H. aspersa* maxima varying from 0.7 to 1.1% [26], 1.5% in *H. aspersa aspersa*, and 1.4% in *H. lucorum* [20]).

Table 2 shows the macro-elements and microelements found in *H. aspersa* Müller powder. The analysis of these results reveals that K (432.47 mg·100 g^−1^) has a significant advantage over the other elements found. Ca was the second-most-prevalent mineral found in the flesh of *H. aspersa* Müller after K, with a value of 482.47 mg·100 g^−1^. Snail flesh has the highest calcium level when compared to other animal products such as milk, eggs, liver, and beef, which have Ca contents of 120, 54, 6, and 7 mg·100 g^−1^, respectively. Ca is implicated in bone and tooth calcification. Its scarcity may therefore have an effect on the structure of bones, causing them to weaken. Ions are required for blood coagulation, as well as nerve and muscle function [29]. The high Ca content of the snail breed tested shows that eating snails may raise Ca in the body, which will have a significant impact on the control of the blood clotting process.

Several investigations demonstrate that Ca content varies significantly, depending on species. Çağıltay et al. [25] found a Ca concentration of 135.7 mg·100 g^−1^ for *H. aspersa,* but in Baby et al. [30], lower values were observed in the *Helix* species (13.6 mg·100 g^−1^). Madejón et al. [31] corroborated this intra-species variability in the species *T. pisana,* finding that, among other minerals, the Ca content showed significant differences based on the location where the snails were taken, as well as their eating patterns. Variations of up to 2000 mg·100 g^−1^ dry weight have been found [31].

It is well-known that Fe helps the oxidation of lipids, proteins, and carbohydrates. *H. aspersa* Müller snail flesh is rich in this element (42.89 ± 0.17 mg·100 g^−1^) compared to conventional animal products such as kidneys, liver, sardines, beef, eggs and milk (6, 11.4, 2.9, 1.9, 2.1 and 0.1 mg·100 g^−1^ respectively) [29]. The obtained results were superior to those of the other authors; for example, the study of Çağıltay et al. [25] reported an average of 17.1 mg·100 g^−1^ in *H. aspersa*, while in the Babalola and Akinsoyinu [11] study, the Fe content was between 15 and 26 mg·100 g^−1^ in *A. marginata*, *A. achatina* and *A. fulica.*

The present research indicated that Zn is abundant in high amounts (13.66 mg·100 g^−1^). This element is involved in a variety of functions in the human system, including adaptation to darkness and night vision [32]. Zn values in *H. pomatia* were reported by Özogul et al. [12] and Drozd et al. [33] to be 1.4 and 1.98 mg·100 g^−1^, respectively. The same authors noted, in *H. aspersa* maxima and *H. aspersa* Müller, concentrations ranging from 2.03 to 2.09 mg·100 g^−1^. As a result, it can be determined that the flesh of *H. aspersa* Müller is a significant source of Zn.

Cu at 0.56 mg·100 g^−1^, was determined to be a highly essential element in snail flesh. This trace element is necessary for the proper functioning of various enzyme systems, including cytochrome oxidase and tyrosinase. Cu catalyzes the oxidation-reduction pathways involved in tissue respiration, when combined with Fe [29]. Furthermore, this flesh includes Mg in the amount of 256.79 ± 4.76 mg·100 g^−1^.

*H. aspersa* Müller flesh has 577.28 mg·100 g^−1^ of phosphorus. In the literature, phosphorus levels have been published [10,11,12,25], however they once again show variation across species as well as within-species variation. With the determination of values between 890 and 1030 mg·100 g^−1^, Madejón et al. [31] revealed this variability, studying snails of the same species that were gathered in several locations.

Total ash and mineral composition is tightly correlated with origin, food, species, life cycle, and seasonal and zonal features, as well as mineral content and bioavailability in the environment [11,12]. The mineral concentration and composition of snail flesh are influenced by all of these variables and different levels of mineral intake; therefore, outcomes can differ significantly, even amongst individuals of the same species, depending on holding circumstances, development, and environmental influences [10,11].

### 2.3. Calorific Content and Nutritional Value

*H. aspersa* Müller dried flesh is a nutrient-rich, low-calorie food. Compared to other meals, a 100 g serving of dried *H. aspersa* Müller flesh provides a modest contribution to the daily required calorie intake, providing only around 76.91 kcal (321.24 kJ). Additionally, it offers 0.59% of the RDI of carbs, 1.16% of the RDI of fat, and 2.10% of the RDI of protein.

According to daily values based on a 2000 calorie diet and a recommended dietary allowance mentioned in European legislation (Table 3) [34], a more acceptable daily intake of 100 g provides about 20.66% of the RDI in protein, 3.64% of the RDI in fat, and 3.20% of the RDI in carbohydrate.

To reach the RDI, 184.98 g of dried *H. aspersa* Müller flesh would be required for calcium, 199.69 g for magnesium, 129.87 g for zinc, 105.26 g for copper, 145.99 g for iron, 185.19 g for manganese, and 121.25 g for phosphorus. Thus, 100 g/d of dry *H. aspersa* Müller flesh offers 77% of the RDI for zinc, 54% of the RDI for calcium, 50% of the RDI for magnesium, 68% of the RDI for iron, 59% of the RDI for copper, and 82% of the RDI for phosphorus.

### 2.4. Fatty Acids Content

The fatty-acid profile of *H. aspersa* Müller is shown in Table 4, an examination of these findings reveals that there are 20 fatty acids in the flesh of *H. aspersa* Müller. The unsaturated fatty acid content was 50.78%, and this ratio was higher than those of saturated fatty acids. The major fatty acids were arachidonic acid (10.67%), palmitic acid (7.11%), stearic acid (16.86%), oleic acid (7.03%), linolenic acid (5.56%), 11.14-Eicosadienoic acid (5.42%), linoleic acid (14.16%), and 34% was composed of saturated fatty acid.

However, 10.33 and 40.45%, respectively, of the total acids were MUFA and PUFA. The main saturated fatty acid was stearic acid (C_18:0_), followed by palmitic acid (C_16:0_) which made up 49.59 and 20.91% of the total saturated fatty acid content, respectively. Oleic acid (C_18:1_) was determined to be the primary monounsaturated fatty acid (approximately 68.05% of all MUFA), while linoleic acid (C_18:2_) was determined to be the primary polyunsaturated fatty acid (about 35.00% of all PUFA).

According to published research in the literature, Milinsk et al. [26] reported that the major fatty acids found in *H. pomatia* were C_16:0_ (6%), C_18:0_ (11%), C_18:1_ (14%), C_18:2_ (20%), and C_20:2_ (11%), while the main fatty acids of *H. memoralis* were C_16:0_ (6%), C_18:0_ (13%), C_18:1_ (15%), C_18:2_ (19%), and C_20:2_ (13%). Özogul et al. [12] discovered in *H. pomatia* 21 fatty acids with 12% C_16:0_, 19% C_18:0_, 7% C_22:0_, 17% C_18:1_, 16% C_18:2_ and 10% C_20:2_.

A helpful metric for assessing the relative nutritional benefits of dietary oils has been proposed, namely, the ω3/ω6 ratio. Numerous studies have linked a greater ratio of ω3/ω6 to improved nutritional value [35]. The daily ω3/ω6 ratio in a healthy human diet should be less than 1:5, according to WHO guidelines [36]. Despite being within the suggested range (0.32), the ω3/ω6 ratio of *H. aspersa* Müller is nevertheless high.

The ω6/ω3 ratio was reported to be, at most, 4.0 [37]; this ratio was lower (3.12) in *H. aspersa* Müller, in which Özogul et al. [12] observed a higher value of 4.94.

The potential advantages of MUFAs in reducing the risk of cardiovascular disease have been studied by many researchers [38]. In this view, Hooper et al. [39] report that PUFAs are crucial in the treatment of autoimmune illnesses, type 2 diabetes, inflammatory ailments, and cardiovascular disease. Psota et al. [40] reiterate the cardioprotective benefits of ω3, while Berbert et al. [41] claims a positive effect of ω3 on rheumatoid arthritis.

From these results, we can conclude that PUFAs are essential to human physiology and should be included in the diet, perhaps in the form of snail flesh, which is a particularly good source of these fatty acids.

### 2.5. Biochemical Analysis

The aqueous extract has an extraction yield of approximately 6.38%. Comprehensive biochemical analysis of the aqueous extract of *H. aspersa* Müller revealed interesting information on its composition. The analysis showed that reducing sugars were present at a concentration of 2.92 ± 0.04 mg·g^−1^. Phenolic compounds, known for their potential health benefits, were present in average quantities of around 1.467 ± 0.077 mg·g^−1^, while the flavonoid content was 0.132 ± 0.002 mg·g^−1^.

On the other hand, these results are consistent with previous research. In one particular study, the crude extract of *H. aspersa* was shown to contain 0.132 mg·mL^−1^ of polyphenols [42]. This crucial finding raises the possibility that HAAE contains an assortment of bioactive compounds which, tantalizingly, could have antioxidant effects. The presence of phenolics and flavonoids, both renowned for their antioxidant properties, underscores the tantalizing prospect that HAAE could harbor a repertoire of molecules capable of mitigating oxidative stress and enhancing overall cellular health.

### 2.6. Antioxidant Activity

The results showed that the total antioxidant activity of HAAE was 9.628 ± 0.010 mg AAE·g^−1^ DM, indicating that the flesh of *H. aspersa* could be a natural source of antioxidants. Other results have been reported by Gogas et al. [43] in *H. aspersa* Müller (0.587–0.715) fed with different protein sources under intensive rearing conditions.

The total antioxidant activity was evaluated by creating a green phosphate complex with an acidic ionic nature; this indicates that HAAE is a good source of natural antioxidants. Indeed, *H. aspersa* Müller could be useful for pharmaceutical applications, and further study of the antioxidant response compounds and their characterization is needed.

The DPPH radical scavenging activity was evaluated by quantifying the inhibition of the DPPH radical by the extract. The effect of HAAE on hydroxyl-radical-induced oxidative damage at different concentrations (200–1000 μg·mL^−1^) was found to be between 36.07 ± 0.5 and 89.35 ± 0.52%. The maximum inhibition was observed at the highest concentration of 1000 μg·mL^−1^ of HAAE, while the scavenging activity of L-ascorbic acid was 89.46% at a concentration of 200 μg·mL^−1^. The reduction in absorbance of the DPPH radical is induced by antioxidants because of the reaction between the antioxidant and the radicals, which results in the recovery of the radical by the donation of hydroxyl.

**Figure 1:**
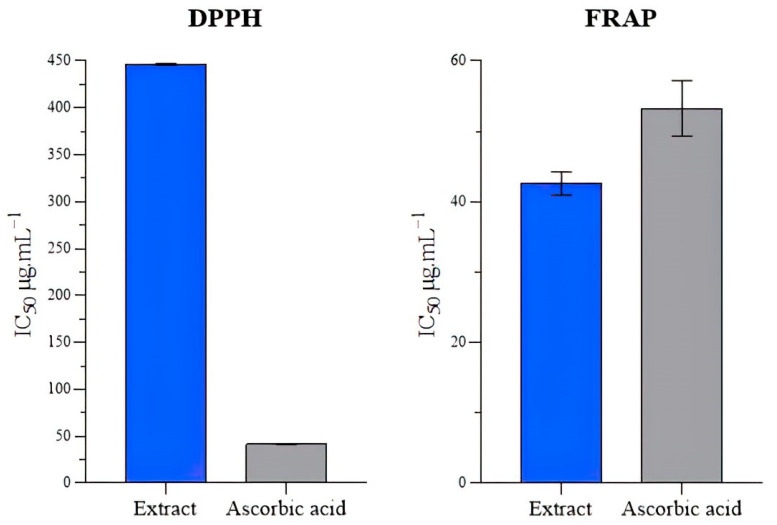
Results of DPPH and FRAP tests of the HAAE and ascorbic acid.

The results showed that the extract had an IC_50_ value of 446.136 ± 0.902 µg·mL^−1^, which was lower than that of ascorbic acid (41.553 ± 0.353 µg·mL^−1^) (Figure 1). Although the extract had a lower antioxidant activity than does ascorbic acid, it still showed potential as a source of natural antioxidants. Comparing this with the previous literature, the methanolic extract of *P. trapezium* showed an IC_50_ value of 4021 µg·mL^−1^ for DPPH radical scavenging activity [44]. The methanolic extract of *B. spinosa* showed an IC_50_ value of 39.43% at 10,000 µg·mL^−1^ [45].

According to Zou et al. [46], the reducing power of the aqueous extract of *H. aspersa* Müller strongly depends on the presence of reducers, which have antioxidant potential by disrupting free radical chains to generate hydrogen atoms. Higher concentrations of antioxidants become pro-oxidants and lead to higher antioxidant activity, so dose dependence can be very significant in the application of antioxidants. However, even at low concentrations, the DPPH radical scavenging capacity of ascorbic acid was superior to that of the tested samples.

In this study, HAAE had potential activity in scavenging hydroxyl radicals at different concentrations. Natural polyphenols generally play a crucial role in natural medicines, and may be responsible for increased free-radical scavenging activity by increasing the levels of phenolic and polyphenolic compounds [47].

The antioxidant activity of the HAAE was measured by quantifying its capacity to transfer an electron to convert Fe^3+^ to Fe^2+^. The quantity of Fe^2+^ can then be determined by detecting the development of Perl’s Prussian blue at 700 nm. The HAAE demonstrated good ferrous-reducing activity in this research, with an IC_50_ value of 42.58 ± 1.64 µg·mL^−1^; however, the reducing power rises with the increasing concentration of the HAAE. The reduction activity was compared to ascorbic acid as a reference antioxidant (53.253 ± 2.277 µg·mL^−1^), and there was a significant difference (*p* ≤ 0.05) between the analyzed extract and ascorbic acid.

The existence of reductions, which have antioxidant potential by disrupting the chain of free radicals by giving a hydrogen atom, determines the ferrous reduction capacity [46]. The chemicals in *H. aspersa* Müller’s water extract may work similarly, providing electrons and interacting with radicals to transform them into more stable products and terminate the chain reaction of free radicals.

Kumaran and Karunakaran [48] found similar findings in the *Phyllanthus* species, in which the lowering ability was proportionate to the amounts. Subavathy and Janet [49] discovered maximum activity at 500 µg·mL^−1^ concentrations of 73.35, 95.36, and 87.5% in *T. brunneus*, *C. annulus*, and *B. spirata*, respectively, and minimum activity at 100 µg·mL^−1^ concentrations of 52.07, 67.21, and 57.51% in *T. brunneus, C. annulus,* and *B. spirata*, respectively. At the maximum quantity (100 µg·mL^−1^) of *O. macrocera,* a greater reducing capability was found [50].

### 2.7. Acute Toxicity

**Figure 2:**
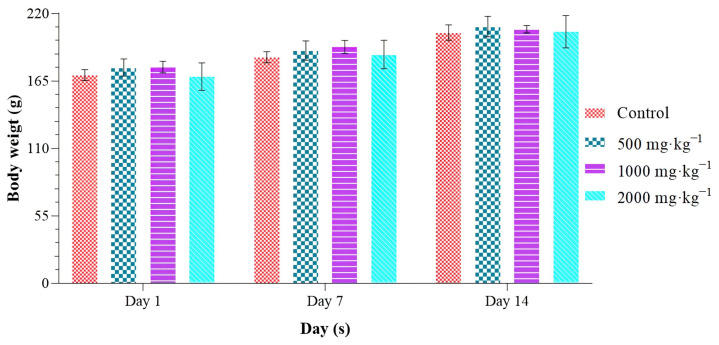
Body-weight development during acute toxicity assessment in mice as a function of time. The values are expressed as mean ± SD (*n* = 5).

The research we conducted on the toxicity of *H. aspersa* Müller flesh is presented in Table 5 and Figure 2. Based on the experiments of oral administration of different doses of HAAE, we noticed that it did not cause any deaths or changes in behavior of rats during the experimental period. Indeed, these trials confirm that the LD_50_ is greater than 2000 mg·kg^−1^. Consequently, no signs of toxicity were observed during the first four hours following administration, with no decreased sensitivity to stimuli (pain and noise), decreased mobility, softening of feces, alteration of behavior, loss of body weight, or death registered for 14 days.

HAAE is classified as a fifth-category substance according to the OECD Globally Harmonized System of Classification and Labelling of Chemicals (GHS), rated as non-toxic by the oral route [51]. According to the Hodge and Sterner [52] scale, the aqueous extract of snail flesh is declared to be non-toxic.

The effects associated with daily oral administration of repeated doses of HAAE were closely scrutinized. However, evaluation of behavioral parameters and weight gain showed no changes in the aforementioned parameters.

Observation of behavior throughout the study period showed that regardless of the dose administered, no behavioral changes were observed during the experimental period. As shown in Figure 2, the body weight of the rats gradually increased over time. However, the body weight of the rats receiving the extract did not show a significant change over time, compared to the controls. These results demonstrate that oral administration of the extract has no effect on the normal growth of rats.

In agreement with the other studies, Danilova [53] found that fresh meat of *H. aspersa* maxima, *H. aspersa* Müller, and *H. pomatia* appeared non-toxic, and that the mice remained healthy throughout the experiment. Kouachi et al. [42] found that at a concentration of 160 mg, rats showed the best weight gain, and the behavior of all animals remained normal. Therefore, *H. aspersa* crude extract was not toxic.

Caetano et al. [27] suggest that the consumption of snails would not incur significant health risks. Moreover, snail meat consumption is not regular, not only because it is a seasonal product, but also because it is not part of the regular consumption of meat at main meals.

Several scientific research efforts have shown that land snails exhibit sensitivity towards the accumulation of heavy metals and other contaminants, making these substances suitable candidates for bio-indicators [10,33,54]. The presence of a significant quantity of heavy metals might potentially result in toxicity among those who ingest these species, provided that the quantities were above the thresholds established by the European Union rules (EU Commission Regulation N° 1881/2006). These limits are set at 1.5, 0.5, and 1.0 mg·kg^−1^ for lead, mercury, and cadmium, respectively. It is important to note that the tests conducted in our study adhered to a meticulously established methodology. The snails used in the experiments were obtained under controlled laboratory conditions, thereby ensuring the lack of any external contamination. An interesting opportunity for further exploration is in the examination of the potential toxicity shown by indigenous snail species inhabiting regions characterized by industrial or agricultural activities.

## 3. Materials and Methods

### 3.1. Snail Harvesting and Laboratory Upkeep

Uninfected *H. aspersa* Müller snails were gathered during the spring season in the Moulay Bousselham region of Morocco. These were immediately moved to the laboratory, where healthy individuals were housed in rectangular plastic boxes (24 × 32 × 12 cm) with a sponge, moist soil, and food (lettuce, carrot, and spinach). Crates holding 50 snails were sprinkled with water every day to keep them wet. This experiment included shells weighing 10.44 ± 3.11 g and measuring 3.35 ± 0.48 cm in length.

We should mention that the snails were treated as part of a scientific experiment that followed animal care guidelines.

### 3.2. Preparation of H. aspersa Müller Flour

150 snails were cleaned with running water, and then stripped of their shells; the visceral masses were eliminated. The feet (with the head) were recovered and cleared of slime with a NaCl solution (1.2%) and were washed with distilled water and dried on absorbent paper. The samples were dried in an oven (60 °C for 48 h), then ground and sieved for recovery.

### 3.3. Proximate Analysis

#### 3.3.1. Moisture Content

The method used for moisture determination was based on the loss of sample mass until a constant mass was obtained at 105 °C [55].

#### 3.3.2. Dry-Matter Content

The determination of the percentage of dry matter (DM) involves placing the sample in an oven at a temperature of 70 °C until a variation of at least 3 mg is obtained between two consecutive weighings performed separately with a 2-h interval [56].

#### 3.3.3. Acidity Measurement

The pH measurement was determined using the method described in [57]. In addition, the determination of acid content was performed by titrating the acidity with a 20% (NaOH) sodium hydroxide solution in the presence of a colored indicator (Phenolphthalein) [55].

#### 3.3.4. Ash Content

Consistent with ISO 936, total ash from snail samples was evaluated using the gravimetric technique at 550 °C. The results were represented as a percentage (g of total ash per 100 g of dry matter) [58].

#### 3.3.5. Protein Content

The AOAC method (928.08) was used to estimate the average total protein in *H. aspersa* Müller flesh [59]. Results were given as grams of protein per 100 g of dry weight. Nitrogen content was converted to protein content using a Jones factor of 6.25.

#### 3.3.6. Lipid Content

The Soxhlet technique was used to calculate the mean lipid content, which was expressed in g·100 g^−1^ wet weight [59].

#### 3.3.7. Total Carbohydrate Content

Following Equation (1), the amount of carbohydrates in the flesh of *H. aspersa* Müller was calculated by deducting the amounts of fat, protein, and ash from the dry-matter value [60].

Carbs (g·100 g^−1^) = DM − (P + A + F) (1)

where DM stands for total dry matter (%), P for protein content (%), A for ash content (%), and F for total fat (lipids contents) (%).

### 3.4. Mineral Composition

The mineral elements were determined in the calcined residue for 16 h at 480 °C using a 1 g test sample. The ash was diluted in concentrated nitric acid (25%) and filtered. This extract was used for mineral determination. The determination of the trace elements studied was carried out by inductively coupled argon plasma atomic emission spectrometry (Rf power 1500 watt, plasma gas flow rate (Ar) 8 L/min, auxiliary gas flow rate (Ar) 0.2 L/min, axial view size, copying and playback time 45 min, and copying time 15 min) were used to determine the trace elements studied. 

Because phosphorus is found in organic molecules, oxidizing the organic material to liberate the phosphorus requires a digestion or calcination procedure. Therefore, using AOAC Method 965.17 [61], the total phosphorus content of *H. aspersa* Müller flesh was determined colorimetrically.

### 3.5. Fatty Acid Composition

A modified version of the transmethylation process from Milinsk et al. [26] was used to create fatty-acid methyl esters. After being placed in screw-capped tubes, the lipid extract sample was combined with n-hexane until the lipid content was dissolved. After adding KOH in methanol ((0.1 M) 2 mol·L^−1^), the mixture was violently agitated. The top layer was transferred to bottles once the layers had been separated.

The fatty-acid methyl esters were analyzed by gas chromatography–mass spectrometry under the following conditions: the injector port temperature was 250 °C., the starting oven temperature was 40 °C, and the temperature was gradually increased at 8 °C·min^−1^ for 18 min until it reached 260 °C. The BR-5ns FS capillary column (30 m × 0.25 mm ID × 0.25 m) was utilized. In undivided mode, the injection volume of helium is 1.0 mL·min^−1^. The whole analysis took 100 min. The mass spectrometry detector (MSD) was set to electronic impact ionization mode, with an ionizing energy of 70 eV and an *m*/*z* scan range of 50 to 500.

The temperature of the ion source was 230 °C, and it then quadrupled to 150 °C. The electron multiplier voltage (EM voltage) was kept at 1100 V above the self-regulatory level, with a 3 min solvent delay.

### 3.6. Antioxidant Activity and Toxicity of the Aqueous Extract of H. aspersa Müller Flesh (HAAE)

#### 3.6.1. Aqueous Extract of *H. aspersa* Müller Preparation

Ten grams of snail flesh flour were mixed with 100 mL of distilled water and subjected to maceration for 48 h. The resulting mixture was filtered using filter paper, and the filtrate volume was evaporated using a rotary evaporator and dried at 60 °C until the weight stabilized. The obtained crystals were crushed using a mortar, and the resulting powder was stored in a sterile glass jar hermetically sealed and kept refrigerated at 4 °C. The extraction yield (EY) was calculated relative to the weight of the dry material using the following equation, Equation (2):

(2) $$\mathrm{EY}=\frac{\mathrm{Obtained}\;\mathrm{extract}\;\mathrm{mass}}{\mathrm{Powder}\;\mathrm{initial}\;\mathrm{mass}}\times 100$$

#### 3.6.2. Determination of Total Phenolic Content and Total Flavonoid Content

The total phenol content of HAAE was determined by the Folin–Ciocalteu method reported by Zargoosh et al. [62], with some modifications. In contrast, the total flavonoid content was estimated using the aluminum trichloride (AlCl_3_) method [63].

#### 3.6.3. Content of Reducing Sugars

The quantification of reducing sugars was based on the use of Di-Nitro-Salicylic Acid as a reagent according to the method of Negrulescu et al. [64].

#### 3.6.4. Antioxidant Activity

- Total antioxidant capacity (TAC)

In this assay, 50 µL of HAAE is mixed with 2000 µL of reagent (0.6 M sulfuric acid, 28 mM sodium phosphate and 4 mM ammonium molybdenum). The reaction mixture was then incubated at 95 °C for 90 min, cooled to room temperature and the absorbance at 695 nm was recorded against a blank. L-ascorbic acid was used as a reference antioxidant. The total antioxidant capacity of the extract was expressed as ascorbic-acid equivalent per gram of dry matter (mg EAA·g^−1^ DM) [65].

- Free radical scavenging activity (DPPH)

The absorption of DPPH radicals at characteristic wavelengths is followed by a decrease in optical density. From a series of tubes, 500 µL of ethanol solutions containing different concentrations of extracts were taken, and 2500 µL of DPPH solution (0.2 mM) was added to all tubes. The contents were shaken well and allowed to react at room temperature for 30 min. The absorbance of the samples was recorded at 517 nm against the control. A negative control was made by mixing 500 µL of methanol and 2500 µL of DPPH solution, and the methanol was used as a blank in parallel with the test sample [66].

The percentage inhibition was calculated as follows in Equation (3):

(3) $$\%\;\mathrm{Activity}\;=\frac{{\mathrm{Abs}}_{\;\mathrm{Cn}}-{\mathrm{Abs}}_{\;\mathrm{Ech}}}{\mathrm{Abs}\;\mathrm{Cn}}\times 100$$

where Abs _Ech_: absorbance of the sample and Abs _Cn_: absorbance of negative control.

- Ferrous-ion-reducing antioxidant power assay (FRAP)

The formation of the ferrous form of the iron/ferricyanide complex was measured by the formation of pearly Prussian blue at 700 nm [67]. 200 µL of extracts of varying concentrations, containing 250 µL of phosphate buffer (0.2 M, pH = 6.6) and 250 µL of potassium ferricyanide (1%, *w*/*v*), were allowed to react at 50 °C for 20 min. Then, 2500 µL of TCA (10%, *w*/*v*) were added and the mixture was centrifuged (10 min, 3000 rpm). 2500 µL supernatant, 500 µL ferric chloride (0.1%, *w*/*v*) and 2500 µL distilled water were mixed. The absorbance was determined at 700 nm relative to negative control. The same procedure was followed for the standard solution of L-ascorbic acid. The reduction of activity can be calculated using the following analysis, Equation (4):

(4) $$\%\;\mathrm{Reduction}\;\mathrm{power}\;=\frac{\mathrm{Abs}\;\mathrm{of}\;\mathrm{sample}\;–\mathrm{Abs}\;\mathrm{of}\;\mathrm{blank}}{\mathrm{Abs}\;\mathrm{of}\;\mathrm{sample}}\times 100$$

where Abs of blank = absorbance without sample and Abs of sample = absorbance of the sample.

### 3.7. Evaluation of Acute Toxicity of the Aqueous Extract of H. aspersa Müller Flesh

The ethical institutional committee for the Faculty of Sciences, University Ibn Tofail, Kenitra, Morocco, approved the protocol. All of the experimental proceedings using laboratory animals followed the Organization for Economic Cooperation and Development (OECD) guidelines 423 [51]. The experiment was conducted on 20 female Wistar albino rats. The behavior of the rats was observed, and the number of deaths over a 14-day period was determined. Prior to the test, the rats were fasted for 6 h and then separated into four groups, each containing 5 females [51]. Three groups were orally administered doses of *H. aspersa* Müller at 500, 1000, and 2000 mg·kg^−1^, respectively, while the control group received distilled water at 10 mL·kg^−1^. Behavioral observation was conducted 1 h after administration.

During the experiment, the rats were provided with hydration and daily food in equal quantities. Additionally, signs of toxicity, such as changes in coat, motility, tremors, changes in body weight (mass), food intake, grooming, respiration, stool appearance, mobility, and respiration, as well as convulsions and deaths, were noted during the few hours after gavage and then daily for 14 days.

### 3.8. Statistical Analysis

The analyses’ results were presented as mean and standard deviation (SD). All experimental data collected were realized in triplicate (*n* = 3). One-way analysis of variance (ANOVA) was used to analyze the obtained data, followed by Duncan’s multiple distribution test. α = 5% was considered to describe the significant level (SPSS Version 20).

## 4. Conclusions

The results clearly show that *H. aspersa* Müller meat has a very high nutritional value, which is originated in its substantial protein content. The notable presence of unsaturated fatty acids in this flesh highlights its nutritional value. A harmonious balance between ω6 and ω3 fatty acids, combined with a favorable alliance between polyunsaturated and monounsaturated fatty acids, lends eminent importance to this remarkable composition. In addition, the abundance of essential minerals such as potassium, calcium, and iron confirms the vital importance of this food resource. Its potential as a rich source of bioactive molecules is undeniably implied by the considerable presence of antioxidants and the diverse range of chemicals detected in its aqueous extract. However, it is essential to note that oral administration of this extract showed no signs of acute toxicity in terms of behavioral effects, and it is equally remarkable that no noticeable disruption of the rats’ body weights was observed following this administration. Thus, using OECD standards, our study allows flesh from *H. aspersa* Müller to be included in the fifth group. It should be stressed, nonetheless, that these findings require more research through extended studies of chronic oral toxicity, cytotoxicity, and histological examinations of certain organs in rats. Such comprehensive studies are imperative for a more profound understanding of the potential benefits and safety aspects associated with the consumption of *H. aspersa* Müller flesh and that of any other species utilized locally.

## Figures and Tables

**Table 1:** Proximate composition of *H. aspersa* Müller.

Parameters	%
Moisture	84.24 ± 1.21
Dry matter	17.62 ± 0.74
Acidity	0.45 ± 0.01
Protein	10.33 ± 0.21
Carbohydrate	2.88 ± 0.04
Fat	2.55 ± 0.00
Ash	1.89 ± 0.05

**Table 2:** Mineral content of *H. aspersa* Müller.

Minerals	mg·100 g^−1^
Calcium (Ca)	432.47 ± 18.43
Potassium (K)	597.11 ± 30.37
Magnesium (Mg)	187.79 ± 0.50
Sodium (Na)	83.10 ± 2.52
Cobalt (Co)	0.06 ± 0.05
Copper (Cu)	0.59 ± 0.09
Iron (Fe)	9.59 ± 0.18
Manganese (Mn)	1.08 ± 0.16
Zinc (Zn)	7.70 ± 0.99
Phosphorus (P)	577.28 ± 7.19

**Table 3:** Energy, macronutrients, and micronutrients described in Regulation 1169/2011: Recommended daily intakes (RDI).

Energy/Nutrient	Reference Intake (per Day)
Energy	8400 kJ (2000 kcal)
Fat	70 g
Carbohydrates	260 g
Sugars	90 g
Proteins	50 g
Calcium (Ca)	800 mg
Magnesium (Mg)	375 mg
Copper (Cu)	1 mg
Iron (Fe)	14 mg
Manganese (Mn)	2 mg
Zinc (Zn)	10 mg
Phosphorus (P)	700 mg

**Table 4:** Fatty acid profile of the *H. aspersa* Müller flesh.

Fatty Acids	%
C_16:0_ Palmitic acid	7.11
C_17:0_ Margaric acid	2.58
C_17:0_ Heptadecanoic acid	1.94
C_18:0_ Stearic acid	16.86
C_20:0_ Arachidic acid	1.05
C_22:0_ Behenic acid	1.71
Octanoic acid	0.75
C_14:0_ Myristic acid	0.56
C_15:0_ Pentadecanoic acid	0.46
C_23:0_ Tricosanoic acid	0.38
Saturated fatty acid SFA	34.00
C_16:1_ Palmetoleic acid	0.60
C_18:1_ Oleic acid (ω9)	7.03
C_20:1_ Eicosanoic acid (ω9)	3.30
C_24:1_ Nervonic acid (ω9)	2.19
Monunsaturated fatty acid MUFA	10.33
C_18:2_ Linoleic acid (ω6)	14.16
C_18:2_ Linoelaidic acid (ω6)	1.70
C_18:3_ Linolenic acid (ω3)	5.56
C_20:2_ 11.14-Eicosadienoic acid (ω11)	5.42
C_20:4_ Arachidonic acid (ω6)	10.67
5.8.11.14.17-Eicosatetraenoic (ω3)	2.94
Polyunsaturated fatty acid PUFA	40.45
Total ω3	8.50
Total ω6	26.53
PUFA/SFA	1.18
PUFA/MUFA	3.92
Not identified	13.03

Results expressed as percentage of total fatty acid methyl esters. SFA: saturated fatty acids, MUFA: monounsaturated fatty acids, PUFA: polyunsaturated fatty acids.

**Table 5:** Acute toxicity evaluation.

Dose	Number of Rats	Signs of Toxicity	Mortality	LD_50_	Category
Control	5	-	0	>2000 mg·kg^−1^	5
500 mg·kg^−1^	5	-	0		
1000 mg·kg^−1^	5	-	0		
2000 mg·kg^−1^	5	-	0		

## Data Availability

Not applicable.

## Sample Availability

Samples of the compounds are available from the authors.
